# Iron Deficiency and Iron Deficiency Anemia: Implications and Impact in Pregnancy, Fetal Development, and Early Childhood Parameters

**DOI:** 10.3390/nu12020447

**Published:** 2020-02-11

**Authors:** Robert T. Means

**Affiliations:** 1 Departments of Internal Medicine, Medical Education, and Pathology, James H. Quillen College of Medicine, East Tennessee State University, Johnson City, TN 37614, USA; meansr@etsu.edu; Tel.: +1-423-439-6499; Fax: +1-423-439-6470; 2 Internal Medicine, Building 2/Room 109, Quillen College of Medicine, East Tennessee State University, Johnson City, TN 37614, USA

**Keywords:** iron deficiency, pregnancy, iron deficiency anemia, laboratory testing, iron supplementation, iron balance

## Abstract

A normal pregnancy consumes 500–800 mg of iron from the mother. Premenopausal women have a high incidence of marginal iron stores or iron deficiency (ID), with or without anemia, particularly in the less developed world. Although pregnancy is associated with a “physiologic” anemia largely related to maternal volume expansion; it is paradoxically associated with an increase in erythrocyte production and erythrocyte mass/kg. ID is a limiting factor for this erythrocyte mass expansion and can contribute to adverse pregnancy outcomes. This review summarizes erythrocyte and iron balance observed in pregnancy; its implications and impact on mother and child; and provides an overview of approaches to the recognition of ID in pregnancy and its management, including clinically relevant questions for further investigation.

## 1. Introduction

Anemia with a hemoglobin (Hb) concentration no lower than 10 g/dL at term, occurs in nearly all pregnancies, and in the majority of cases reflects a physiologic process (discussed below) rather than a deficiency state or underlying hematologic disorder [1]. Significant anemia in pregnancy (defined as a Hb concentration <11 g/dL in the first trimester or <10 g/dL in the second and third trimesters) occurs with a prevalence ranging between 2% and 26%, depending upon the population studied [2,3,4]. Anemia is a major contributor to maternal and fetal morbidity and mortality, particularly in less developed countries [2,3,4,5,6]. Of the pathologic causes of anemia in pregnancy, anemia due to iron deficiency (IDA) is the most common, particularly in more developed countries, where contributions from other anemia-producing disorders such as malaria or hemoglobinopathies are less significant [7,8,9].

Anemia in pregnancy, and particularly IDA, is both a long-standing scholarly interest and an element of my practice as a hematologist. In preparing for this review, PubMed searches using the terms “pregnancy” and “iron deficiency” were performed. A date range was not specified, but the focus was on papers 2015 and later. Specific searches for subtopics included “hepcidin”, and “guidelines”. Approximately 71 reports, reviews, or studies new to the author were identified through this process.

## 2. Physiologic Anemia of Pregnancy

The effects of ID on red cell production occur in the context of what is usually called the *physiologic anemia of pregnancy*. This is a phenomenon that is conserved across mammalian species [10,11], and it is hypothesized that the physiologic anemia of pregnancy serves the purpose of enhancing placental perfusion by reducing maternal blood viscosity and facilitating oxygen and nutrient delivery to the fetus by expanding the erythrocyte mass [12]. Beginning approximately the sixth week of pregnancy, the plasma volume increases disproportionately to the erythrocyte mass, reaching a maximum value at approximately 24 weeks’ gestation. At maximum, the plasma volume is 40%50% higher than at the start of pregnancy [13].

Since the parameters used to identify anemia in clinical practice (the hematocrit (Hct), the blood Hb concentration, and the circulating erythrocyte count) are expressed as concentrations based on whole blood volume, the expanded plasma volume causes them to decrease and hence produces “anemia”. While Hb concentration, Hct, and to a lesser degree erythrocyte count, are the anemia indicators used in practice, these parameters are only surrogates for the actual definition of anemia: a reduction in erythrocyte mass per unit body weight [14]. By this criterion, the physiologic anemia of pregnancy is not actually anemia: a 15%25% increase in the erythrocyte mass occurs in pregnancy but is concealed by the dilutional effect of the increase in plasma volume [15]. This is driven by an increase in serum erythropoietin concentrations during the late second and early third trimesters [16] and is facilitated or potentially limited by iron availability. Pregnant women using iron supplementation have a greater increase in erythrocyte mass than women not using supplemental iron [1], and women with compromised iron stores at outset of pregnancy will have a limited increase in erythrocyte mass. The upper limit of erythrocyte mass increase in the presence of adequate iron is, however, regulated through erythropoietin control and is not raised by increased iron availability: pregnant women in the Bantu tribe, who have both an iron-rich traditional diet and a genetic predisposition to increased dietary iron absorption, do not increase Hb concentration or Hct with supplementation [17,18].

**Figure 1:**
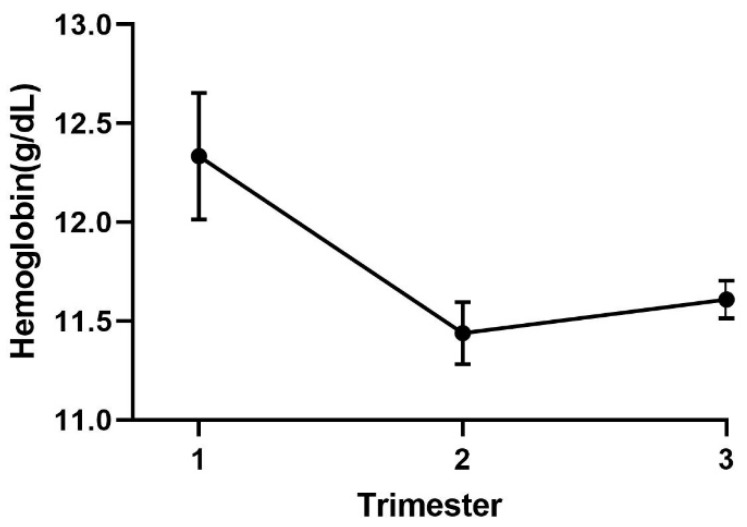
Hemoglobin concentrations of healthy women during pregnancy (mean ± standard deviation. Data combined from references [19,20,21].

As a result of the reset balance between plasma volume and erythrocyte mass, it is generally considered that a Hb concentration <11 g/dL in the late first trimester and <10 g/dL in the second and third trimesters should be investigated for a cause other than the physiologic anemia of pregnancy [19]. Figure 1 shows hemoglobin concentrations by trimester based on reports from Norway [19], Jamaica [20], and China [21].

Maternal plasma volume generally decreases during the final weeks of pregnancy, and consequently the Hct, Hb, and circulating erythrocyte count increase [13]. The maternal blood volume generally returns to prepregnancy levels within one to six weeks after delivery and maternal erythropoiesis increases late in gestation and returns to normal by about one month after delivery [22].

## 3. Iron Balance: An Overview

A detailed discussion of the regulation of iron balance is outside the scope of this review, and the reader is referred to recent reviews [23,24,25]. Iron content in the human body is carefully regulated and is normally maintained at about 40 mg/kg in women and about 50 mg/kg in men. Since humans are unable to excrete excess iron in a regulated manner, iron balance is controlled at the levels of iron absorption by enterocytes in the duodenum, and of iron mobilization from liver parenchyma and macrophages. These processes are regulated by hepcidin, a small peptide produced in the liver. Hepcidin binds to a cellular iron export protein, ferroportin, causing its internalization. When hepcidin levels are increased, iron is retained in enterocytes or macrophages and is not available for red cell production. When hepcidin is decreased, either because of ID or by increased erythropoiesis, absorbed iron in the enterocytes or stored iron in macrophages are mobilized into the circulation [23,24].

Absorption of dietary iron is also affected by the iron source and duodenal conditions, such as pH. The proportion of iron absorbed from heme iron and non-food sources such as iron salts or saccharates is approximately 10%15% of elemental iron, while less than 2% of elemental iron from vegetable sources is absorbed. ID may double the percent iron absorption from any given source [26].

Iron is transported from the enterocyte to the plasma iron transport protein transferrin. The amount of iron bound to transferrin and in circulation is approximately 0.2 mg/kg [27] under normal circumstances. Storage iron resides in macrophages of the spleen, bone marrow, or liver, and in liver parenchymal cells (5–6 mg/kg in women, 10–12 mg/kg in men). Macrophage iron is largely derived from recycling of senescent erythrocytes while liver parenchymal cells receive or release iron from or to transferrin [27]. The largest pool of iron in the body is in circulating erythrocytes and erythroid precursors in the bone marrow (approximately 28 mg/kg in women and 32 mg/kg in men). Nearly all of this erythrocyte iron is in the form of Hb [27].

## 4. Iron Requirements during Pregnancy

It is generally considered that a normal singleton pregnancy carried to term requires a transfer of 500–800 mg of maternal iron [28]. It is estimated that the demand for absorbed iron increases from 0.8 mg/day in early pregnancy to 7.5 mg/day in late pregnancy, with an average requirement over the entire course of pregnancy of 4.4 mg/day [29]. In a study of healthy pregnant women in Denmark, the 5th percentile Hb value of subjects receiving supplementation with 66 mg elemental iron/day was consistently higher than that of the subjects receiving placebo. Differences were small in the first trimester (0.1 mg/dL Hb higher) and gradually increased into the second (0.1–0.4 mg/dL Hb higher) and third trimesters (0.3–0.9 mg/dL Hb higher) and the postpartum period (1 mg/dL Hb higher) [19]. The relatively small difference in the first trimester likely reflects a high incidence of ID or marginal iron stores in both groups and the steadily increasing gap reflects the increasing iron requirements of pregnancy that are not being met in the placebo group. In a large review of premenopausal women, only 20% had presumed iron reserves of >500 mg (defined as a serum ferritin concentration >70 µg/L), and would potentially be able to go through pregnancy without iron supplementation [30]. This is consistent with an earlier study in which women who received or did not receive iron supplementation during pregnancy underwent bone marrow evaluation after delivery. Only 16% of women not supplemented with oral iron had stainable iron in bone marrow aspirates after delivery at term, although their mean Hb concentration (10.9 g/dL) was in the expected range for the third trimester [1].

## 5. ID in Premenopausal Women

ID reflects depleted iron stores. Although the “gold standard” for ID is the absence of stainable reticuloendothelial iron on a bone marrow specimen, in clinical practice it is usually defined by surrogate laboratory markers such as a low serum ferritin or a decreased percentage of transferrin saturation by iron [31]. Values for these parameters are discussed below. 

The frequency of ID in pregnancy reflects not only the iron requirements of pregnancy but also the high frequency of ID among premenopausal women. Specific estimates vary depending on the population studied and the specific parameter used to define ID. Differing diagnostic criteria for ID in pregnancy are discussed below. In a recent study reported from the USA defining ID by either low serum ferritin (<30 µg/L) or low transferrin saturation (<19%), 42% of unselected women in the first trimester who were not anemic met laboratory criteria by for ID [32]. Frequency of ID may also differ by patient population. Using a serum ferritin cutoff of 20–30 µg/L to indicate ID or minimal iron stores [32,33,34,35], at least 25% of women age 15–39 participating in the US Third National Nutrition and Health examination Survey (NHANES) [36], were iron deficient (Table 1). The subsets of African-American and non-Latina white women resembled the overall survey population, but 50% of Latina women in this age range were iron deficient. In a review of studies from Europe, 40%55% of premenopausal women were reported as iron deficient [35].

For the most part, ID in premenopausal women results primarily from an imbalance between nutritional iron intake and *physiologic* blood loss through menses or previous pregnancy. Decreased dietary iron intake can contribute to this imbalance, as in women following a vegetarian or vegan diet who do not use an iron supplement, or who have iron malabsorption [37,38]. Menstrual blood loss can represent 25–50 mg iron per cycle [33] depending upon the patient’s Hct/Hb concentration and the individual pattern of menstruation, and can be substantially greater in patients with menorrhagia [39]. This is distinct from the situation for men and for post-menopausal women, in whom ID is a consequence of blood loss from a *pathologic* lesion, most commonly in the gastrointestinal tract, and where an endoscopic investigation for a source of blood loss is required [40]. However, pregnant women may require evaluation for a site of pathologic blood loss if the clinical situation suggests it.

## 6. Evaluation for ID and IDA in Pregnancy

ID can exist without anemia when iron stores are absent but the ongoing iron deficit is not sufficiently severe to produce a decreased Hb concentration. This state is much more common in healthy women than in healthy men. In a study defining the normal range for serum soluble transferrin receptor concentrations, ID with normal blood counts was seen in 9.5% of healthy adult women volunteers (premenopausal and postmenopausal) but only 1.5% of men [41]. As noted earlier, in a study of non-anemic women in the first trimester of pregnancy, 42% were iron deficient [32]. ID without anemia is frequently a transient stage; the clinical significance of ID without anemia is a subject of active research [42].

**Figure 2:**
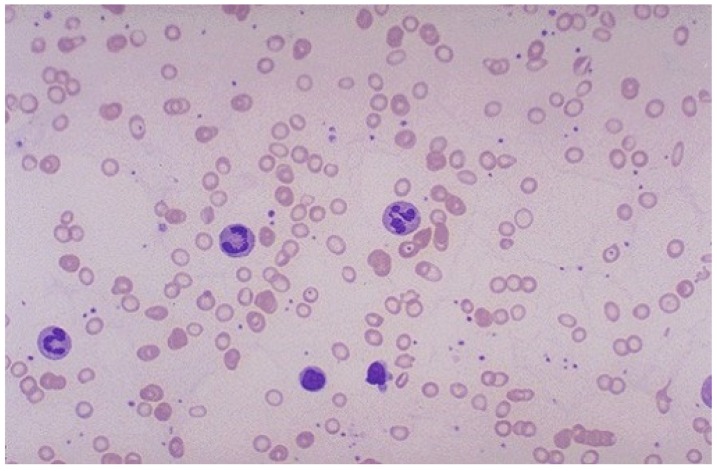
Peripheral blood film, iron deficiency anemia. (1000×, oil).

IDA develops when iron intake is insufficient to meet continuing demands for red cell production, and follows stages that have been well defined for many years [43]. The initial stage is referred to as iron deficient erythropoiesis, or sometimes iron-restricted erythropoiesis. This term is also used to describe erythropoiesis in circumstances such as the anemia of chronic disease/inflammation where cytokine-driven hepcidin production impairs iron mobilization despite sufficient storage iron [44]. In the initial stages of iron deficient erythropoiesis, Hb concentration/Hct begin a gradual decline, but erythrocytes remain morphologically normal. The biochemical tests noted in Table 2 (serum iron concentration, total iron binding capacity (TIBC), transferrin concentration, and ferritin concentration) are decreased at this point. As noted above, if iron intake is sufficient to maintain the Hb/Hct and daily losses, the patient may remain in a steady state of iron deficient erythropoiesis. Otherwise, as unmet demands for iron continue, a decline in Hb of 1–2 mg/dL occurs (which may still be within the Hb normal range), the average erythrocyte size (reported by electronic cell counters as the mean corpuscular volume, MCV) begins to decline. Shortly thereafter, the concentration of Hb in the erythrocyte (mean corpuscular Hb concentration, MCHC) also begins to decline. This pattern is outlined in Table 2. The speed with which these changes occur and progress to IDA depends on the ongoing iron deficit. This process of decreased MCV (microcytosis) and decreased MCHC (hypochromia) leads to the characteristic morphologic appearance of IDA [43]. (Figure 2).

Although a number of biochemical tests for the assessment of iron status are widely available (Table 2), their interpretation and use are affected by a variety of circumstances. Serum ferritin concentration is a marker of reticuloendothelial iron stores, and a serum ferritin concentration that is below the normal range is the most specific biochemical indicator of ID. A number of studies suggest that it is the most effective single test for ID screening in pregnancy [45,46]. However, there is disagreement as to what constitutes a serum ferritin concentration diagnostic of ID in women [47,48]. This reflects the high frequency of asymptomatic ID among healthy women from whom population-based laboratory values are derived. As an example, the lower limit of normal for serum ferritin concentration from an international reference laboratory is 11 µg/L in women but 24 µg/L in men (www.mayocliniclabs.com). In contrast, studies correlating serum ferritin concentrations with bone marrow iron stains suggests that the ferritin value indicative of absent or significantly reduced marrow iron stores is approximately 30 µg/ L iron stores, without relation to subject sex [34,49]. A recent systematic review of serum ferritin thresholds for ID in pregnancy reported that most studies used values of 12–15 µg/L but that thresholds ranged as high as 30 µg/L. Rationales for the choice of a threshold were rarely provided, and the conclusion of the authors was that standardization is required [47,50]. Table 3 lists thresholds used in various studies and guidelines. Lower ferritin thresholds are associated with significantly decreased sensitivity for ID with a minimal increase in sensitivity. It has been estimated that a ferritin threshold at 30 µg/L is 98% specific and 92% sensitive for ID, while a serum ferritin threshold of 10 µg/L is only 25% sensitive [32]. Although a low serum ferritin is always indicative of ID, serum ferritin is an acute phase reactant that may be increased out of proportion to iron stores by infection, inflammation, liver disease, malignancy, or other conditions. In such circumstances, ID may be present with normal or mildly increased serum ferritin concentration [51] This is typically not an issue in otherwise healthy young women but may be a confounding factor in more complex patients. Methods for adjusting serum ferritin based on markers of inflammation such as C-reactive protein have been proposed [52], although in most cases the use of additional tests (discussed below) will identify ID when it is suspected in a patient with normal serum ferritin concentration. 

As noted earlier, iron for erythropoiesis circulates bound to transferrin. Transferrin may be directly measured and expressed as a protein concentration or expressed functionally as TIBC (quantity of bound iron per volume of serum or plasma). A detailed discussion of the relationship between these parameters is outside the scope of this review [58,59]. From a clinical perspective, they serve the same diagnostic purpose, and clinical laboratories frequently report one or the other. Transferrin and TIBC are elevated in uncomplicated ID [60], but may be decreased by inflammation or malnutrition [61]. Similarly, a decreased serum or plasma iron concentration may reflect decreased iron stores or may reflect impaired iron mobilization due to inflammation and mediated by hepcidin in most cases [62,63]. The availability of iron for erythropoiesis (as distinct from iron stores estimated by serum ferritin) is assessed by the percent of transferrin/TIBC saturation by iron. A decreased transferrin saturation (particularly when the transferrin concentration/TIBC is normal or increased) correlates with other indicators of ID in pregnancy [64], and can indicate ID in patients with a normal ferritin concentration in whom ID is suspected on a clinical or statistical basis.

An increased concentration of serum soluble transferrin receptor (sTfR) is associated with ID but also with increased erythropoiesis from congenital hemoglobinopathies, other causes of hemolysis, or ineffective erythropoiesis. It is not increased or decreased by inflammation [31,41]. In pregnancy, demonstration of an elevated serum sTfR concentration, particularly in a patient with a normal or elevated serum ferritin concentration suspected to be elevated out of proportion to iron stores, confirms ID [65]. The specificity of sTfR for ID may be enhanced by expressing it as the ratio of sTfR to the log value of serum ferritin [66]. However, the sTfR ratio does not appear to be more effective than serum ferritin concentration in more general circumstances [67].

The role of hepcidin as the primary mediator of iron homeostasis has been noted earlier. Hepcidin levels are increased in the anemia of inflammation/chronic disease and decreased in ID, and can be used to distinguish these two syndromes, both of which are associated with hypoferremia [68]. Measurement of hepcidin for clinical purposes is not widely available at present but may become so in the next few years. Hepcidin appears to be an effective indicator of ID in pregnancy [69,70], but its specific role in management remains to be determined [71].

## 7. Impact of ID during Pregnancy

Assessing the impact of maternal ID on pregnancy course and on early childhood development is more complex than it might appear at first. ID is more common in economically and socially disadvantaged populations [72], and these challenges may contribute independently to complications in pregnancy and early childhood development. In the case of IDA, questions may arise as to whether any negative effect is a general consequence of anemia or is specific to ID [73]. In one recent report in women who were infected with schistosomiasis during pregnancy, maternal IDA appear to predict child ID at six months of age [74]. ID may also be a marker for other nutritional issues. In a study from Pakistan, underweight women in early pregnancy had lower serum ferritin concentrations than either normal body weight or overweight mothers [75].

ID can contribute to impaired cognitive development in early childhood [76], and the opportunity to intervene during pregnancy and avoid this complication is appealing from a public health perspective. However, it is not entirely clear that maternal iron supplementation during pregnancy can reverse effects of maternal ID during pregnancy on neurodevelopment [77,78]. Two recent systematic reviews found no evidence that iron supplementation during pregnancy improved neurodevelopment in offspring [76,79]. The specific frequency of ID in the populations studied may have contributed to the absence of a statistically significant effect: benefits may have been confined to patients who were iron deficient or had marginal iron stores at the time of treatment [79,80]. From a standpoint of newborn anemia/iron status, the evidence appears more clear: studies spanning 60 years that consistently support the concept that maternal ID typically does not reduce fetal iron supply. The fetus appears to be prioritized by the biology of gestation as a recipient of iron and the nutrients required for Hb synthesis. As a result, the Hb concentration of infants born to mothers with IDA is usually normal for infant age and values for biochemical markers of iron status (such as serum iron, transferrin saturation, and serum ferritin) observed in the newborn appear to be unrelated to those observed in the mother [81,82,83,84,85,86,87,88]. However, serum ferritin concentration in cord blood at the time of delivery seems to predict the serum ferritin concentration in the first two years of life [89]. The availability of newer approaches to evaluating iron homeostasis may lead to changes in this paradigm. A report by Lee and colleagues indicates a higher prevalence of newborn anemia and markers of decreased iron availability (decreased cord blood ferritin relative to reported values, increased serum sTfR) in children born to iron-deficient adolescent mothers [90]. Further investigation in this population is certainly warranted, particularly to identify the prevalence of maternal factors independently associated with newborn anemia, such as maternal obesity or tobacco use [91]. While sTfR is increased in ID, it is also a marker of erythropoietic activity, raising the possibility that the cord blood ferritin was relatively low due to increased mobilization of iron stores for erythropoiesis. The elevated erythropoietin levels observed could also be consistent with this interpretation [90]. 

Evidence for effects of maternal ID on the course of pregnancy is clearer. ID leads to placenta hypertrophy [92], and severe maternal ID appears to increase the risks of premature delivery, low birth weight, and infant death [93]. In at least one study, IDA in the first trimester was associated with low birthweight, but development of ID later in pregnancy was not [94]. However, it is important to consider that maternal ID may be a marker for food insecurity or lack of prenatal care, which may have similar effects. It is also a challenge to distinguish effects of anemia generally from effects specific to ID [73]. In the case of the mother, hematologic benefits of iron supplementation are less disputed [95,96]. In a review of published reports, routine iron supplementation decreased the frequency of anemia at term by 73% and the frequency of IDA by 67% [97,98]. 

Table 4 summarizes results from a Cochrane Database systematic review comparing outcomes of daily iron supplementation to placebo or no iron supplementation [71,96].

## 8. Approach to Iron Administration in Pregnancy

At present, neither the US Preventive Services Task Force nor the American College of Obstetricians and Gynecologists (ACOG) take a position on routine iron supplementation in pregnancy [53,99], and society guidelines in the UK recommend against it. Both the ACOG and the UK guidelines recommend screening for anemia as a surrogate for detecting ID. The recommended standards at which anemia should be investigated from both societies are generally consistent with the earlier discussion in this review of the expected degree of anemia from the physiologic anemia of pregnancy: 11.0 g/dL in the first and third trimesters, and 10.5 g/dL in the second trimester (ACOG), or 11.0 g/dL in the first trimester and 10.5 g/dL in subsequent trimesters (UK) [53,54,55]. Both guidelines suggest a trial of iron as the initial step, with subsequent investigation for other causes if there is an insufficient response. This approach poses a risk of missing individuals who are iron deficient but not anemic as well as the early stages of ID. It has been suggested, based upon cross-sectional studies of reproductive age women who are not pregnant, that a hemoglobin threshold of 12.8 g/dL (higher than the World Health Organization standard of 12.0 g/dL) is a more appropriate cut off for identifying women at risk for ID [100]. Neither the ACOG nor UK guidelines recommend routine screening with iron studies, but the UK guidelines recommend measuring serum ferritin in women perceived to be at high risk for ID, even if they are not anemic [54,55]. The most effective approach to anticipating and managing ID risk in pregnancy is a critical topic for future research. (See Section 9, below)

There are two general approaches to iron supplementation in pregnant women who are not anemic. These are selective supplementation, typically guided by laboratory values or by patient demographics in high-risk areas; and routine or universal supplementation. One well-described approach to selective supplementation is based on estimation of iron stores by serum ferritin. When the serum ferritin is greater than 70 µg/L, iron stores are considered adequate to support pregnancy and no supplementation is given. When serum ferritin is less than 30 µg/L, iron stores are considered absent or nearly absent, and the patient is treated with 80–100 mg elemental iron/day orally. Women whose ferritin values are between these points receive low-dose supplementation of 30–40 mg/day [101]. A recent systematic review supports the concept that intermittent iron supplementation in pregnancy (2–3 times weekly, as opposed to daily) is as effective as daily supplementation, and associated with fewer side effects and presumably, higher compliance [102].

By those criteria, more than 75% of the women participating in the Third NHANES study overall (Table 1) would require supplementation at some level, and more than 90% of Latina women would require supplementation. For this reason, many physicians utilize routine or universal iron supplementation in all pregnant women.

An alternate approach to selective supplementation has been proposed using hepcidin as the indicator of early ID and a need for therapy. A recent report comparing a hepcidin-guided supplementation approach to a universal prophylaxis approach showed similar patient outcomes in both groups [71]. This study was performed in a high-risk population in Gambia, and this approach may have different outcomes in other venues.

One reason to avoid routine supplementation in the less developed world is concern that iron supplementation will increase risk of infection with iron dependent microorganisms and parasites, including malaria. A study in Papua New Guinea found that the benefits of iron supplementation on maternal anemia and birthweight exceeded potential risk, although the benefits were most pronounced in patients who had some degree of ID [80]. In general, iron supplementation is considered low risk [53], and an iron supplement of 65 mg elemental iron mg/day beginning at or before 20 weeks’ gestation generally is adequate to prevent ID during pregnancy [30]. However, one of the arguments against routine iron supplementation, particularly in the less-developed world, is that benefits on infant neurocognitive development (as distinct from benefits on maternal anemia and iron stores) have not been demonstrated clearly, as was discussed earlier [79]. 

In keeping with the UK and ACOG guidelines, investigation for an etiology of anemia would occur if Hb were below the levels described. At this point, the focus moves beyond supplementation (which could be regarded as providing the additional iron required for gestation to a person with adequate iron stores) to the treatment of IDA. The objective in the treatment of IDA is correction of anemia and also repletion of absent iron stores. If initiated early in pregnancy, therapy will need to accommodate the 500–800 mg of iron that will be transferred to the newborn as well as maintaining the maternal Hb/Hct and repleting iron stores. A reasonable approach to therapy is to provide 60–100 mg of elemental iron per day. A variety of oral iron preparations are available and patient preference and, in some cases, considerations of the financial cost to the patient, can govern choices. Traditionally, ID was treated with oral iron three times daily. Subsequent studies investigating the interaction of oral iron therapy with the hepcidin axis led to the recognition that the hepcidin increment caused by therapeutic doses of iron salts or saccharates decreases absorption for approximately 24 h, implying that once daily oral iron therapy is as effective or more effective than the traditional twice or three times daily dosing [103]. Failure to respond to oral iron should lead to a re-assessment of iron status (as described Section 6.) This would be to address problems with iron absorption leading to a poor response or (more commonly) lack of compliance with iron therapy, but also to consider other potential etiologies of anemia in pregnancy. Oral iron therapy for IDA in pregnancy should continue until the Hb/Hct and MCV are in the normal range, and until the serum ferritin has also returned to a solidly normal value (certainly higher than 30 µg/L and probably higher than 50 µg/L) indicating adequate iron stores.

Most pregnant patients are able to tolerate oral iron, particularly when given once daily or on an intermittent schedule. However, if the patient is unresponsive to oral iron, or unable/unwilling to take iron orally, intravenous iron therapy is safe and effective [104]. An advantage of intravenous iron therapy is that it corrects Hb/Hct and iron stores concurrently and rapidly. Recent systematic reviews indicate that intravenous iron therapy in pregnancy allows more complete achievement of desired Hb concentrations [105,106]. A number of intravenous iron preparations are available, with different dosing schedules, and a detailed discussion of these is outside the scope of the current review. In most cases, the total dose to be administered intravenously is 1000–1500 mg elemental iron. 

In the absence of gastrointestinal signs or symptoms, endoscopic evaluation of the gastrointestinal tract is unlikely to identify a lesion accounting for blood loss in premenopausal women with ID, and can be deferred safely in favor of a trial of iron replacement [107]. As noted earlier, failure to respond to iron therapy should prompt evaluation for either ongoing sources of blood loss if there is persisting evidence of ID, or consideration of other etiologies of anemia.

## 9. ID in Pregnancy: Areas for Further Research

Although ID in pregnancy is a common syndrome for which effective diagnosis and therapy exists, there remain many opportunities for further research to enhance management and understanding of pathogenesis. A number of these opportunities have been noted in this review. Areas of particular importance include the following:

1. What is the most effective screening approach? While guidelines from specialty societies recommend screening for anemia as a surrogate for the detection of ID, specific assessment of iron status is restricted to high risk subsets of patients. Should specific testing for iron status replace or complement screening for anemia in all patients? If not, what is the appropriate Hb/Hct level II most effectively detect ID?
2. In identifying ID in pregnancy, what is the optimal serum ferritin threshold? Most studies and recommendations favor measurement of serum ferritin concentration as either the recommended initial assessment or as a reasonable choice for initial assessment. Thresholds of serum ferritin concentration for the identification of ID in pregnancy range from 10 µg/L to 30 µg/L. should the standard for ID be the lower limit of the population ferritin range (10–15 µg/L) in women, or should it be a value supported by correlation with bone marrow iron status or with some other factor such as sTfR concentration?
3. Is there a role for universal supplementation? If not, what are the parameters that will guide supplementation? Should they be guided by serum ferritin concentration, transferrin saturation, sTfR, or hepcidin? If a stratified/guided approach to iron supplementation in pregnancy is proposed, what are its benefits and costs compared to universal supplementation, looking at parameters of maternal and fetal/newborn l outcomes as well as iron status?
4. What is the role of hepcidin in the diagnosis of ID in pregnancy and in its management? Hepcidin provides the potential for a mechanistic, biologically based approach to the assessment of ID in pregnancy as opposed to various surrogate measures of iron stores or availability. Is hepcidin more useful in diagnosis and management than other, more standard, iron parameters such as ferritin? What are the limitations around availability and cost-effectiveness?
5. Is there a benefit from maternal iron supplementation/anemia correction for newborn neurocognitive development? As noted in the review, it is unclear that maternal iron supplementation during pregnancy as a beneficial effect on newborn neurocognitive development. Should studies to address this focus only on iron deficient mothers, in whom the potential for benefit may be highest? If such studies demonstrate a benefit, how can it be optimized?
6. What is the most cost- and outcome-effective way to deliver iron supplementation/replacement and to perform screening in resource-poor settings? Mothers with access to a high level of prenatal care have additional options for diagnosis and management of ID, such as laboratory testing for serum ferritin or intravenous iron therapy if there is a poor response to oral iron. Mothers in resource-poor environments (which could include individuals with limited healthcare access in more developed nations) may be limited to Hb/Hct testing and oral iron preparations. What is the optimal way provide treatment of this common clinical issue in a way that recognizes economic limitations but also maximizes favorable outcomes?

## Figures and Tables

**Table 1:** Serum ferritin concentrations (µg/L) for women ages 15–39. (Calculated from Third National Nutrition and Health Examination Survey (NHANES) [36]).

	25th Percentile	50th Percentile	75th Percentile	90th Percentile
All women	20.1	36.0	58.8	92.0
Non-Latina white women	22.1	37.7	59.7	89.0
African American women	18.4	35.9	68.5	117.3
Latina women	15.0	24.7	50.5	62.0

**Table 2:** Pattern of laboratory abnormalities in the development of iron deficient erythropoiesis and IDA.

Parameter	Iron-Deficient Erythropoiesis	IDA
Hb	Normal but may be decreasing	Decreased
Hct	Normal but may be decreasing	Decreased
MCV	Low-normal to decreased	Decreased
MCHC	Low-normal to decreased	Decreased
Serum iron concentration	Decreased	Decreased
Serum transferrin concentration	Increased	Increased
Serum TIBC	Increased	Increased
Serum transferrin or TIBC saturation	<20%	<15%
Serum ferritin concentration	Decreased	Decreased
Serum soluble transferrin receptor concentrations (sTfR)	Increased	Increased

Normal values for specific parameters may differ based on laboratory definitions and population norms. See text for discussion.

**Table 3:** Reported serum ferritin thresholds for the diagnosis of ID in pregnancy.

Year of Publication	Source	Report Type	Ferritin Threshold
2008	USA	Society guideline [53]	10 µg/L
2019	UK	Society guideline [54,55]	30 µg/L
2016	France	Registry study [35,56]	15 µg/L
2017	Europe (15 countries)	Literature survey [35]	30 µg/L
2019	Norway	Population study [57]	15 µg/L

Guidelines were identified using PubMed and the website of the America College of Obstetricians and Gynecologists. Other representative studies were selected from PubMed using the search terms “iron deficiency”, “ferritin”, “pregnancy” since 2015.

**Table 4:** Maternal and newborn outcomes of daily iron supplementation compared to placebo or no supplementation. Summary of a systematic review [96].

	Difference Observed (Number of Studies)	No Difference Observed (Number of Studies)
Mother	↓ Frequency of anemia at term (11)	Frequency of maternal infection (1)
	↓ Frequency of IDA at term (6)	Frequency of maternal mortality (2)
	↓ Frequency of ID at term (7)	Frequency of iron side effects (11)
Newborn	↓Frequency of low birthweight (11)	Neonatal death (4)
	↓ Frequency of preterm delivery (13)	Placental malaria (2)
	↑ Birthweight (15)	

## References

[1] de Leeuw N.K., Lowenstein L., Hsieh Y.S. (1966). Iron deficiency and hydremia in normal pregnancy. Medicine.

[2] Levy A., Fraser D., Katz M., Mazor M., Sheiner E. (2005). Maternal anemia during pregnancy is an independent risk factor for low birthweight and preterm delivery. Eur. J. Obstet. Gynecol. Reprod. Biol..

[3] Adebisi O.Y., Strayhorn G. (2005). Anemia in pregnancy and race in the United States: Blacks at risk. Fam. Med..

[4] Xiong X., Buekens P., Fraser W.D., Guo Z. (2003). Anemia during pregnancy in a Chinese population. Int. J. Gynaecol. Obstet..

[5] Kozuki N., Lee A.C., Katz J., Child Health Epidemiology Reference Group (2012). Moderate to severe, but not mild, maternal anemia is associated with increased risk of small-for-gestational-age outcomes. J. Nutr..

[6] Brabin B.J., Hakimi M., Pelletier D. (2001). An analysis of anemia and pregnancy-related maternal mortality. J. Nutr..

[7] Mettananda S., Suranjan M., Fernando R., Dias T., Mettananda C., Rodrigo R., Perera L., Gibbons R., Premawardhena A., Higgs D. (2018). Anaemia among females in child-bearing age: Relative contributions, effects and interactions of alpha- and beta-thalassaemia. PLoS ONE.

[8] Huynh B.T., Cottrell G., Cot M., Briand V. (2015). Burden of malaria in early pregnancy: A neglected problem?. Clin. Infect. Dis..

[9] Chen C., Grewal J., Betran A.P., Vogel J.P., Souza J.P., Zhang J. (2018). Severe anemia, sickle cell disease, and thalassemia as risk factors for hypertensive disorders in pregnancy in developing countries. Pregnancy Hypertens..

[10] West C.A., Sasser J.M., Baylis C. (2016). The enigma of continual plasma volume expansion in pregnancy: Critical role of the renin-angiotensin-aldosterone system. Am. J. Physiol. Renal Physiol..

[11] Robeck T.R., Nollens H.H. (2013). Hematological and serum biochemical analytes reflect physiological challenges during gestation and lactation in killer whales (*Orcinus orca*). Zoo Biol..

[12] Stangret A., Skoda M., Wnuk A., Pyzlak M., Szukiewicz D. (2017). Mild anemia during pregnancy upregulates placental vascularity development. Med. Hypotheses.

[13] de Haas S., Ghossein-Doha C., van Kuijk S.M., van Drongelen J., Spaanderman M.E. (2017). Physiological adaptation of maternal plasma volume during pregnancy: A systematic review and meta-analysis. Ultrasound Obstet. Gynecol..

[14] Crispin P.J., Sethna F., Andriolo K. (2019). Red cell and reticulocyte parameters for the detection of iron deficiency in pregnancy. Clin. Lab..

[15] Horowitz K.M., Ingardia C.J., Borgida A.F. (2013). Anemia in pregnancy. Clin. Lab. Med..

[16] Milman N., Graudal N., Nielsen O.J., Agger A.O. (1997). Serum erythropoietin during normal pregnancy: Relationship to hemoglobin and iron status markers and impact of iron supplementation in a longitudinal, placebo-controlled study on 118 women. Int. J. Hematol..

[17] Gerritsen T., Walker A.R. (1954). The effect of habitually high iron intake on certain blood values in pregnant Bantu women. J. Clin. Investig..

[18] Gordeuk V.R. (2002). African iron overload. Semin. Hematol..

[19] Milman N., Byg K.E., Agger A.O. (2000). Hemoglobin and erythrocyte indices during normal pregnancy and postpartum in 206 women with and without iron supplementation. Acta Obstet. Gynecol. Scand..

[20] James T.R., Reid H.L., Mullings A.M. (2008). Are published standards for haematological indices in pregnancy applicable across populations: An evaluation in healthy pregnant Jamaican women. BMC Pregnancy Childbirth.

[21] Shen C., Jiang Y.M., Shi H., Liu J.H., Zhou W.J., Dai Q.K., Yang H. (2010). A prospective, sequential and longitudinal study of haematological profile during normal pregnancy in Chinese women. J. Obstet. Gynaecol..

[22] Choi J.W., Pai S.H. (2001). Change in erythropoiesis with gestational age during pregnancy. Ann. Hematol..

[23] Camaschella C., Pagani A., Nai A., Silvestri L. (2016). The mutual control of iron and erythropoiesis. Int. J. Lab. Hematol..

[24] Pagani A., Nai A., Silvestri L., Camaschella C. (2019). Hepcidin and anemia: A tight relationship. Front. Physiol..

[25] Means R.T. (2013). Hepcidin and iron regulation in health and disease. Am. J. Med. Sci..

[26] Taylor P., Martinez-Torres C., Leets I., Ramirez J., Garcia-Casal M.N., Layrisse M. (1988). Relationships among iron absorption, percent saturation of plasma transferrin and serum ferritin concentration in humans. J. Nutr..

[27] Means R.T., Singh A.K. (2016). Red blood cell function and disrders of iron metabolism. Scoentific American Medicine.

[28] Milman N., Bergholt T., Byg K.E., Eriksen L., Graudal N. (1999). Iron status and iron balance during pregnancy. A critical reappraisal of iron supplementation. Acta Obstet. Gynecol. Scand..

[29] Milman N. (2008). Prepartum anaemia: Prevention and treatment. Ann. Hematol..

[30] Milman N., Graudal N., Agger A.O. (1995). Iron status markers during pregnancy. No relationship between levels at the beginning of the second trimester, prior to delivery and post partum. J. Intern. Med..

[31] Means R.T., Allen J., Sears D.A., Schuster S.J. (1999). Serum soluble transferrin receptor and the prediction of marrow aspirate results in a heterogeneous group of patients. Clin. Lab. Haematol..

[32] Auerbach M., Abernathy J., Juul S., Short V., Derman R. (2019). Prevalence of iron deficiency in first trimester, nonanemic pregnant women. J. Mater. Fetal Neonatal Med..

[33] Yokoi K. (2014). Estimation of iron requirements for women by numerical analysis of population-based data from the National Health and Nutrition Surveys of Japan 2003–2007. J. Trace Elem. Med. Biol..

[34] North M., Dallalio G., Donath A.S., Melink R., Means R.T. (1997). Serum transferrin receptor levels in patients undergoing evaluation of iron stores:correlation with other parameters, and observed versus predicted results. Clin. Lab. Haematol..

[35] Milman N., Taylor C.L., Merkel J., Brannon P.M. (2017). Iron status in pregnant women and women of reproductive age in Europe. Am. J. Clin. Nutr..

[36] Hollowell J.G., van Assendelft O.W., Gunter E.W., Lewis B.G., Najjar M., Pfeiffer C. (2005). Hematological and iron-related analytes--reference data for persons aged 1 year and over: United States, 1988–1994. Vital Health Stat. Data Natl. Health Surv..

[37] Sebastiani G., Herranz Barbero A., Borras-Novell C., Alsina Casanova M., Aldecoa-Bilbao V., Andreu-Fernandez V., Pascual Tutusaus M., Ferrero Martinez S., Gomez Roig M.D., Garcia-Algar O. (2019). The effects of vegetarian and vegan diet during pregnancy on the health of mothers and offspring. Nutrients.

[38] Haslam N., Lock R.J., Unsworth D.J. (2001). Coeliac disease, anaemia and pregnancy. Clin. Lab..

[39] Napolitano M., Dolce A., Celenza G., Grandone E., Perilli M.G., Siragusa S., Carta G., Orecchioni A., Mariani G. (2014). Iron-dependent erythropoiesis in women with excessive menstrual blood losses and women with normal menses. Ann. Hematol..

[40] Dubois R.W., Goodnough L.T., Ershler W.B., Van Winkle L., Nissenson A.R. (2006). Identification, diagnosis, and management of anemia in adult ambulatory patients treated by primary care physicians: Evidence-based and consensus recommendations. Curr. Med. Res. Opin..

[41] Allen J., Backstrom K.R., Cooper J.A., Cooper M.C., Detwiler T.C., Essex D.W., Fritz R.P., Means R.T., Meier P.B., Pearlman S.R. (1998). Measurement of soluble transferrin receptor in serum of healthy adults. Clin. Chem..

[42] McLaren G.D., Skikine B.S., Means R.T. (2019). Iron deficiency without anemia. Nutritional Anemia: Scientific Principles, Clinical Practice, and Public Health.

[43] Hillman R.S., Finch C.A. (1974). Red Cell Manual.

[44] Brugnara C. (2003). Iron deficiency and erythropoiesis: New diagnostic approaches. Clin. Chem..

[45] Byg K.E., Milman N., Hansen S., Agger A.O. (2000). Serum ferritin is a reliable, non-invasive test for iron status in pregnancy: Comparison of ferritin with other iron status markers in a longitudinal study on healthy pregnant women. Hematology.

[46] Crispin P., Stephens B., McArthur E., Sethna F. (2019). First trimester ferritin screening for pre-delivery anaemia as a patient blood management strategy. Transfus. Apheresis Sci..

[47] Daru J., Allotey J., Pena-Rosas J.P., Khan K.S. (2017). Serum ferritin thresholds for the diagnosis of iron deficiency in pregnancy: A systematic review. Transfus. Med..

[48] Daru J., Colman K., Stanworth S.J., De La Salle B., Wood E.M., Pasricha S.R. (2017). Serum ferritin as an indicator of iron status: What do we need to know?. Am. J. Clin. Nutr..

[49] Milman N., Pedersen N.S., Visfeldt J. (1983). Serum ferritin in healthy Danes: Relation to marrow haemosiderin iron stores. Dan. Med. Bull..

[50] Casonato A., Galletta E., Sarolo L., Daidone V. (2018). Type 2N von Willebrand disease: Characterization and diagnostic difficulties. Haemophilia.

[51] Cunietti E., Ciari M.M., Monti M., Engaddi I., Berlusconi A., Neri M.C., De Luca P. (2004). Distortion of iron status indices by acute inflammation in older hospitalized patients. Arch. Gerontol. Geriatr..

[52] Suchdev P.S., Williams A.M., Mei Z., Flores-Ayala R., Pasricha S.R., Rogers L.M., Namaste S.M. (2017). Assessment of iron status in settings of inflammation: Challenges and potential approaches. Am. J. Clin. Nutr..

[53] American College of Obstetrics & Gynecology (2008). ACOG Practice Bulletin No. 95: Anemia in pregnancy. Obstet. Gynecol..

[54] Pavord S., Daru J., Prasannan N., Robinson S., Stanworth S., Girling J. (2019). UK guidelines on the management of iron deficiency in pregnancy. Br. J. Haematol..

[55] Pavord S., Myers B., Robinson S., Allard S., Strong J., Oppenheimer C. (2012). UK guidelines on the management of iron deficiency in pregnancy. Br. J. Haematol..

[56] Harvey T., Zkik A., Auges M., Clavel T. (2016). Assessment of iron deficiency and anemia in pregnant women: An observational French study. Womens Health.

[57] Naess-Andresen M.L., Eggemoen A.R., Berg J.P., Falk R.S., Jenum A.K. (2019). Serum ferritin, soluble transferrin receptor, and total body iron for the detection of iron deficiency in early pregnancy: A multiethnic population-based study with low use of iron supplements. Am. J. Clin. Nutr..

[58] Gottschalk R., Wigand R., Dietrich C.F., Oremek G., Liebisch F., Hoelzer D., Kaltwasser J.P. (2000). Total iron-binding capacity and serum transferrin determination under the influence of several clinical conditions. Clin. Chim. Acta.

[59] Kasvosve I., Delanghe J. (2002). Total iron binding capacity and transferrin concentration in the assessment of iron status. Clin. Chem. Lab. Med..

[60] Sharma J.B., Bumma S.D., Saxena R., Kumar S., Roy K.K., Singh N., Vanamail P. (2016). Cross sectional, comparative study of serum erythropoietin, transferrin receptor, ferritin levels and other hematological indices in normal pregnancies and iron deficiency anemia during pregnancy. Eur. J. Obstet. Gynecol. Reprod. Biol..

[61] Tomkins A. (2003). Assessing micronutrient status in the presence of inflammation. J. Nutr..

[62] Ganz T., Nemeth E. (2009). Iron sequestration and anemia of inflammation. Semin. Hematol..

[63] Guida C., Altamura S., Klein F.A., Galy B., Boutros M., Ulmer A.J., Hentze M.W., Muckenthaler M.U. (2015). A novel inflammatory pathway mediating rapid hepcidin-independent hypoferremia. Blood.

[64] Byg K.E., Milman N., Ole Agger A. (2000). Correlations between iron status markers during normal pregnancy in women with and without Iron supplementation. Hematology.

[65] Akinsooto V., Ojwang P.J., Govender T., Moodley J., Connolly C.A. (2001). Soluble transferrin receptors in anaemia of pregnancy. J. Obstet. Gynaecol..

[66] Beguin Y. (2003). Soluble transferrin receptor for the evaluation of erythropoiesis and iron status. Clin. Chim. Acta.

[67] Yang Z., Dewey K.G., Lonnerdal B., Hernell O., Chaparro C., Adu-Afarwuah S., McLean E.D., Cohen R.J., Domellof M., Allen L.H. (2008). Comparison of plasma ferritin concentration with the ratio of plasma transferrin receptor to ferritin in estimating body iron stores: Results of 4 intervention trials. Am. J. Clin. Nutr..

[68] Tussing-Humphreys L., Pusatcioglu C., Nemeth E., Braunschweig C. (2012). Rethinking iron regulation and assessment in iron deficiency, anemia of chronic disease, and obesity: Introducing hepcidin. J. Acad. Nutr. Diet..

[69] Zaman B., Rasool S., Jasim S., Abdulah D. (2019). Hepcidin as a diagnostic biomarker of iron deficiency anemia during pregnancy. J. Matern. Fetal Neonatal Med..

[70] Abioye A.I., Aboud S., Premji Z., Etheredge A.J., Gunaratna N.S., Sudfeld C.R., Noor R.A., Hertzmark E., Spiegelman D., Duggan C. (2019). Hemoglobin and hepcidin have good validity and utility for diagnosing iron deficiency anemia among pregnant women. Eur. J. Clin. Nutr..

[71] Bah A., Muhammad A.K., Wegmuller R., Verhoef H., Goheen M.M., Sanyang S., Danso E., Sise E.A., Pasricha S.R., Armitage A.E. (2019). Hepcidin-guided screen-and-treat interventions against iron-deficiency anaemia in pregnancy: A randomised controlled trial in The Gambia. Lancet Glob. Health.

[72] Bodnar L.M., Scanlon K.S., Freedman D.S., Siega-Riz A.M., Cogswell M.E. (2001). High prevalence of postpartum anemia among low-income women in the United States. Am. J. Obstet. Gynecol..

[73] Ellman L.M., Vinogradov S., Kremen W.S., Poole J.H., Kern D.M., Deicken R.F., Brown A.S. (2012). Low maternal hemoglobin during pregnancy and diminished neuromotor and neurocognitive performance in offspring with schizophrenia. Schizophr. Res..

[74] Abioye A.I., McDonald E.A., Park S., Ripp K., Bennett B., Wu H.W., Pond-Tor S., Sagliba M.J., Amoylen A.J., Baltazar P.I. (2019). Maternal anemia type during pregnancy is associated with anemia risk among offspring during infancy. Pediatr. Res..

[75] Riaz M., Shaikh F., Fawwad A., Hakeem R., Shera A.S., Hitman G.A., Bhowmik B., do Vale Moreira N.C., Basit A., Hussain A. (2018). Maternal nutrition during early pregnancy and cardiometabolic status of neonates at birth. J. Diabetes Res..

[76] Janbek J., Sarki M., Specht I.O., Heitmann B.L. (2019). A systematic literature review of the relation between iron status/anemia in pregnancy and offspring neurodevelopment. Eur. J. Clin. Nutr..

[77] Rioux F.M., Belanger-Plourde J., Leblanc C.P., Vigneau F. (2011). Relationship between maternal DHA and iron status and infants’ cognitive performance. Can. J. Diet. Pract. Res..

[78] Larson L.M., Phiri K.S., Pasricha S.R. (2017). Iron and cognitive development: What Is the evidence?. Ann. Nutr. Metab..

[79] Jayasinghe C., Polson R., van Woerden H.C., Wilson P. (2018). The effect of universal maternal antenatal iron supplementation on neurodevelopment in offspring: A systematic review and meta-analysis. BMC Pediatr..

[80] Verhoef H., Mwangi M.N., Cerami C., Prentice A.M. (2019). Antenatal iron supplementation and birth weight in conditions of high exposure to infectious diseases. BMC Med..

[81] Lukens J.N. (1978). Neonatal haematological abnormalities associated with maternal disease. Clin. Haematol..

[82] Caramihai E., Karayalcin G., Aballi A.J., Lanzkowsky P. (1975). Leukocyte count differences in healthy white and black children 1 to 5 years of age. J. Pediatr..

[83] Lanzkowsky P. (1962). Hematologic values in healthy infants and children in three racial groups in Cape Town. J. Pediatr..

[84] Lanzkowsky P. (1961). The influence of maternal iron-deficiency anaemia on the haemoglobin of the infant. Arch. Dis. Child..

[85] Shott R.J., Andrews B.F. (1972). Iron status of a medical high-risk population at delivery. Am. J. Dis. Child..

[86] Woodruff C.W., Bridgeforth E.B. (1953). Relationship between the hemogram of the infant and that of the mother during pregnancy. Pediatrics.

[87] van Eijk H.G., Kroos M.J., Hoogendoorn G.A., Wallenburg H.C. (1978). Serum ferritin and iron stores during pregnancy. Clin. Chim. Acta.

[88] Harthoorn-Lasthuizen E.J., Lindemans J., Langenhuijsen M.M. (2001). Does iron-deficient erythropoiesis in pregnancy influence fetal iron supply?. Acta Obstet. Gynecol. Scand..

[89] Hay G., Refsum H., Whitelaw A., Melbye E.L., Haug E., Borch-Iohnsen B. (2007). Predictors of serum ferritin and serum soluble transferrin receptor in newborns and their associations with iron status during the first 2 y of life. Am. J. Clin. Nutr..

[90] Lee S., Guillet R., Cooper E.M., Westerman M., Orlando M., Kent T., Pressman E., O’Brien K.O. (2016). Prevalence of anemia and associations between neonatal iron status, hepcidin, and maternal iron status among neonates born to pregnant adolescents. Pediatr. Res..

[91] Ru Y., Pressman E.K., Guillet R., Katzman P.J., Bacak S.J., O’Brien K.O. (2018). Predictors of anemia and iron status at birth in neonates born to women carrying multiple fetuses. Pediatr. Res..

[92] Huang A., Zhang R., Yang Z. (2001). Quantitative (stereological) study of placental structures in women with pregnancy iron-deficiency anemia. Eur. J. Obstet. Gynecol. Reprod. Biol..

[93] Marti A., Pena-Marti G., Munoz S., Lanas F., Comunian G. (2001). Association between prematurity and maternal anemia in Venezuelan pregnant women during third trimester at labor. Arch. Latinoam. Nutr..

[94] Turgeon O’Brien H., Santure M., Maziade J. (2000). The association of low and high ferritin levels and anemia with pregnancy utcome. Can. J. Diet. Pract. Res..

[95] Abraha I., Bonacini M.I., Montedori A., Di Renzo G.C., Angelozzi P., Micheli M., Germani A., Carloni D., Scaccetti A., Palmieri G. (2019). Oral iron-based interventions for prevention of critical outcomes in pregnancy and postnatal care: An overview and update of systematic reviews. J. Evid. Based Med..

[96] Pena-Rosas J.P., De-Regil L.M., Dowswell T., Viteri F.E. (2012). Daily oral iron supplementation during pregnancy. Cochrane Database Syst. Rev..

[97] Yakoob M.Y., Bhutta Z.A. (2011). Effect of routine iron supplementation with or without folic acid on anemia during pregnancy. BMC Public Health.

[98] Mahomed K. (2000). Iron supplementation in pregnancy. Cochrane Database Syst. Rev..

[99] Siu A.L. (2015). Screening for iron deficiency anemia and iron supplementation in pregnant women to improve maternal health and birth outcomes: U.S. Preventive Services Task Force recommendation statement. Ann. Intern. Med..

[100] Sekhar D.L., Kunselman A.R., Chuang C.H., Paul I.M. (2017). Optimizing hemoglobin thresholds for detection of iron deficiency among reproductive-age women in the United States. Transl. Res..

[101] Milman N. (2006). Iron prophylaxis in pregnancy—General or individual and in which dose?. Ann. Hematol..

[102] Pena-Rosas J.P., De-Regil L.M., Gomez Malave H., Flores-Urrutia M.C., Dowswell T. (2015). Intermittent oral iron supplementation during pregnancy. Cochrane Database Syst. Rev..

[103] Moretti D., Goede J.S., Zeder C., Jiskra M., Chatzinakou V., Tjalsma H., Melse-Boonstra A., Brittenham G., Swinkels D.W., Zimmermann M.B. (2015). Oral iron supplements increase hepcidin and decrease iron absorption from daily or twice-daily doses in iron-depleted young women. Blood.

[104] Auerbach M. (2018). Commentary: Iron deficiency of pregnancy—A new approach involving intravenous iron. Reprod. Health.

[105] Lewkowitz A.K., Gupta A., Simon L., Sabol B.A., Stoll C., Cooke E., Rampersad R.A., Tuuli M.G. (2019). Intravenous compared with oral iron for the treatment of iron-deficiency anemia in pregnancy: A systematic review and meta-analysis. J. Perinatol..

[106] Sultan P., Bampoe S., Shah R., Guo N., Estes J., Stave C., Goodnough L.T., Halpern S., Butwick A.J. (2019). Oral vs intravenous iron therapy for postpartum anemia: A systematic review and meta-analysis. Am. J. Obstet. Gynecol..

[107] Park D.I., Ryu S.H., Oh S.J., Yoo T.W., Kim H.J., Cho Y.K., Sung I.K., Sohn C.I., Jeon W.K., Kim B.I. (2006). Significance of endoscopy in asymptomatic premenopausal women with iron deficiency anemia. Dig. Dis. Sci..

